# Self-Administration of an Endogenous Cannabinoid 2-Arachidonoylglycerol in Experimentally Naïve Rats

**DOI:** 10.4172/2329-6488.1000e126

**Published:** 2015-10-17

**Authors:** Takato Hiranita

**Affiliations:** Division of Neurotoxicology, National Center for Toxicological Research (NCTR), U.S. Food and Drug Administration (FDA), 3900 NCTR Road, Jefferson, AR 72079-9501, USA

A recent study by Dr. Maria Antonietta De Luca demonstrated intravenous (IV) self-administration responding (nose-poking) of an endogenous compound in an experimentally naïve, adult rat species [1]. Surprisingly, response-dependent changes of visual stimulus were not presented in the study when the compound was injected. This finding is very unique since there is few if any endogenous compounds that have been reported to maintain self-administration responding above vehicle levels through an IV route of administration (i.e. not intracranial self-injections) in rodent species. Further, it is also likely that a phytocannabinoid (−)-trans-Δ9-tetrahydrocannabinol (Δ9-THC, Figure 1), a primary psychoactive constituent in marijuana, is not an effective reinforcer in rat [2,3] and rhesus monkey species [4-6] relative to standard drugs of abuse [7].

The use of marijuana has been legalized in two states of the U.S as of today. Despite high effectiveness of Δ9-THC in experimentally naïve squirrel monkeys [8], Δ9-THC has been reported to fail to maintain IV self-administration responding above vehicle levels in rats [2,3] and rhesus monkeys [4-6]. On the other hand, there continues to be an increase in the abuse and non-medical use of a number of ‘designer’ drugs [9-11]. Among these drugs are synthetic cannabinoids that are frequently found in many K2/Spice preparations [9-11]. Several synthetic cannabinoids have been found to maintain IV self-administration responding in experimentally naïve rats [12-16], and mice [17-20]. For endocannabinoids, only anandamide has been demonstrated to maintain IV self-administration responding in a squirrel monkey species [21]. However, the sample size was only one to draw any conclusion [21]. Using IV drug self-administration procedures in squirrel monkeys, another endocannabinoid 2-arachidonoylglycerol (Figure 1) has been shown to substitute for anandamide or (-)-nicotine [22]. These findings may suggest the reinforcing effects of endocannabinoid in rats. Importantly, the IV self-administration of endocannabinoid anandamide in an experimentally naïve squirrel monkey [21] and of synthetic cannabinoids in experimentally naïve rats [13,14] and mice [17,19,20] occurred when response-dependent changes of visual stimulus were presented. Despite the low effectiveness of phytocannabinoid Δ9-THC in rats as a positive reinforcer and a lack of response-dependent changes of visual stimulus, the endocannabinoid 2-arachidonoylglycerol maintained IV self-administration responding above vehicle levels in all six of six experimentally naïve rats assessed (i.e., 100% of rats assessed) [1]. The finding should be appreciated because endogenous monoamine dopamine, an important neurotransmitter for induction of reinforcing effects of stimulants [23,24], failed to maintain IV self-administration responding above vehicle levels when substituted for (-)-cocaine in rats [25]. Further, a dopamine D2-like agonist quinpirole has been found to fail to induce IV self-administration responding above vehicle levels in experimentally naïve rats even when a response-dependent injection-paired visual stimulus was presented [26,27]. In addition, (-)-nicotine has been found to fail to induce IV self-administration responding above vehicle levels in experimentally naïve rats when an injection-paired visual stimulus was absent [28]. Finally a synthetic cannabinoid WIN 55,212-2 was reinforcing in only a maximum of 85.7% of experimentally naïve rats assessed (=12/14) among a range of several injection doses [13]. Thus it appears that the endocannabinoid 2-arachidonoylglycerol is a relatively effective positive reinforcer in rats.

As mentioned above, the abuse of synthetic cannabinoids is increasing [9,10]. Despite the low effectiveness of a phytocannabinoid Δ9-THC in a rat species [2,3], Dr. De Luca found a relatively high capacity of an endocannabinoid 2-arachidonoylglycerol to induce reinforcing effects in experimentally naïve rats [1]. The self-administration model of 2-arachidonoylglycerol may be useful to study pharmacology of endocannabinoids. In addition, the finding may lead to further development of medications for cannabinoid abuse in humans using a rat species.

## Figures and Tables

**Figure 1:**
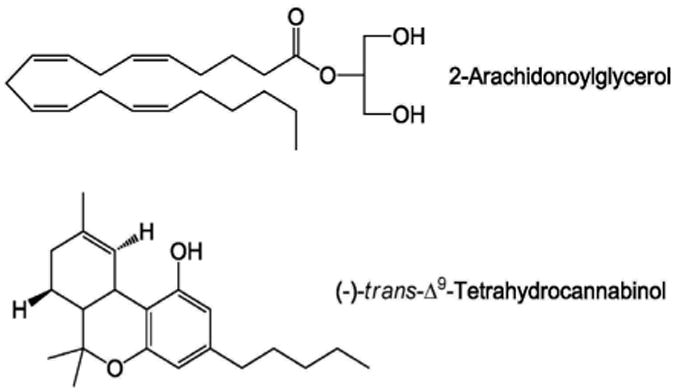
Chemical structures of 2-arachidonoylglycerol (1,3-dihydroxy-2-propanyl (5Z,8Z,11Z,14Z)-5,8,11,14-eicosatetraenoate) and (−)-trans-Δ9-tetrahydrocannabinol [(−)-(6aR,10aR)-6,6,9-trimethyl-3-pentyl-6a,7,8,10a-tetrahydro-6H-benzo[c]chromen-1-ol].

## References

[1] De Luca MA, Valentini V, Bimpisidis Z, Cacciapaglia F, Caboni P (2014). Endocannabinoid 2-Arachidonoylglycerol Self-Administration by Sprague-Dawley Rats and Stimulation of in vivo Dopamine Transmission in the Nucleus Accumbens Shell. Front Psychiatry.

[2] Cha HJ, Lee KW, Song MJ, Hyeon YJ, Hwang JY (2014). Dependence Potential of the Synthetic Cannabinoids JWH-073, JWH-081, and JWH-210: In Vivo and In Vitro Approaches. Biomol Ther (Seoul).

[3] Lefever TW, Marusich JA, Antonazzo KR, Wiley JL (2014). Evaluation of WIN 55,212-2 self-administration in rats as a potential cannabinoid abuse liability model. Pharmacol Biochem Behav.

[4] Mansbach RS, Nicholson KL, Martin BR, Balster RL (1994). Failure of Delta(9)-tetrahydrocannabinol and CP 55,940 to maintain intravenous self-administration under a fixed-interval schedule in rhesus monkeys. Behav Pharmacol.

[5] Harris RT, Waters W, McLendon D (1974). Evaluation of reinforcing capability of delta-9-tetrahydrocannabinol in rhesus monkeys. Psychopharmacologia.

[6] Carney JM, Uwaydah IM, Balster RL (1977). Evaluation of a suspension system for intravenous self-administration studies of water-insoluble compounds in the rhesus monkey. Pharmacol Biochem Behav.

[7] Hiranita T, Kohut SJ, Soto PL, Tanda G, Kopajtic TA (2014). Preclinical efficacy of N-substituted benztropine analogs as antagonists of methamphetamine self-administration in rats. J Pharmacol Exp Ther.

[8] Justinova Z, Panlilio LV, Moreno-Sanz G, Redhi GH, Auber A (2015). Effects of Fatty Acid Amide Hydrolase (FAAH) Inhibitors in Non-Human Primate Models of Nicotine Reward and Relapse. Neuropsychopharmacology.

[9] Seely KA, Lapoint J, Moran JH, Fattore L (2012). Spice drugs are more than harmless herbal blends: A review of the pharmacology and toxicology of synthetic cannabinoids. Prog Neuropsychopharmacol Biol Psychiatry.

[10] Papanti D, Schifano F, Botteon G, Bertossi F, Mannix J (2013). “Spiceophrenia”: A systematic overview of “spice”-related psychopathological issues and a case report. Hum Psychopharmacol.

[11] Baumann MH, Solis E, Watterson LR, Marusich JA, Fantegrossi WE (2014). Baths salts, spice, and related designer drugs: The science behind the headlines. J Neurosci.

[12] De Luca MA, Bimpisidis Z, Melis M, Marti M, Caboni P (2015). Stimulation of *in vivo* dopamine transmission and intravenous self-administration in rats and mice by JWH-018, a Spice cannabinoid. Neuropharmacology.

[13] Fattore L, Cossu G, Martellotta CM, Fratta W (2001). Intravenous self-administration of the cannabinoid CB1 receptor agonist WIN 55,212-2 in rats. Psychopharmacology (Berl).

[14] Spano MS, Fattore L, Cossu G, Deiana S, Fadda P (2004). CB1 receptor agonist and heroin, but not cocaine, reinstates cannabinoid-seeking behaviour in the rat. Br J Pharmacol.

[15] Flores Á, Maldonado R, Berrendero F (2014). The hypocretin/orexin receptor-1 as a novel target to modulate cannabinoid reward. Biol Psychiatry.

[16] Lecca D, Cacciapaglia F, Valentini V, Di Chiara G (2006). Monitoring extracellular dopamine in the rat nucleus accumbens shell and core during acquisition and maintenance of intravenous WIN 55,212-2 self-administration. Psychopharmacology (Berl).

[17] Navarro M, Carrera MR, Fratta W, Valverde O, Cossu G (2001). Functional interaction between opioid and cannabinoid receptors in drug self-administration. J Neurosci.

[18] Ledent C, Valverde O, Cossu G, Petitet F, Aubert JF (1999). Unresponsiveness to cannabinoids and reduced addictive effects of opiates in CB1 receptor knockout mice. Science.

[19] Mendizábal V, Zimmer A, Maldonado R (2006). Involvement of kappa/dynorphin system in WIN 55,212-2 self-administration in mice. Neuropsychopharmacology.

[20] Martellotta MC, Cossu G, Fattore L, Gessa GL, Fratta W (1998). Self-administration of the cannabinoid receptor agonist WIN 55,212-2 in drug-naive mice. Neuroscience.

[21] Justinova Z, Solinas M, Tanda G, Redhi GH, Goldberg SR (2005). The endogenous cannabinoid anandamide and its synthetic analog R(+)-methanandamide are intravenously self-administered by squirrel monkeys. J Neurosci.

[22] Justinová Z, Yasar S, Redhi GH, Goldberg SR (2011). The endogenous cannabinoid 2-arachidonoylglycerol is intravenously self-administered by squirrel monkeys. J Neurosci.

[23] Hiranita T, Mereu M, Soto PL, Tanda G, Katz JL (2013). Self-administration of cocaine induces dopamine-independent self-administration of sigma agonists. Neuropsychopharmacology.

[24] Hiranita T, Soto PL, Tanda G, Kopajtic TA, Katz JL (2013). Stimulants as specific inducers of dopamine-independent Ï*f* agonist self-administration in rats. J Pharmacol Exp Ther.

[25] Pilla M, Perachon S, Sautel F, Garrido F, Mann A (1999). Selective inhibition of cocaine-seeking behaviour by a partial dopamine D3 receptor agonist. Nature.

[26] Collins GT, Woods JH (2007). Drug and reinforcement history as determinants of the response-maintaining effects of quinpirole in the rat. J Pharmacol Exp Ther.

[27] Collins GT, Woods JH (2009). Influence of conditioned reinforcement on the response-maintaining effects of quinpirole in rats. Behav Pharmacol.

[28] Palmatier MI, Evans-Martin FF, Hoffman A, Caggiula AR, Chaudhri N (2006). Dissociating the primary reinforcing and reinforcement-enhancing effects of nicotine using a rat self-administration paradigm with concurrently available drug and environmental reinforcers. Psychopharmacology (Berl).

